# Association between stress hyperglycemia ratio and all-cause mortality in critically ill patients with atrial fibrillation: insights from a MIMIC-IV study

**DOI:** 10.3389/fendo.2024.1412159

**Published:** 2024-08-23

**Authors:** Lin Liu, Zhanfang Zhu, Kai Yu, Wei Zhang, Jie Pu, Ying Lv, Zhiguo Tang, Fuqiang Liu, Shasha Liu

**Affiliations:** ^1^ Department of Cardiology, Shaanxi Provincial People’s Hospital, Xi’an, China; ^2^ Department of Internal Medicine, Xi’an Jiaotong University Hospital, Xi’an, China; ^3^ Department of Cardiology, Pucheng County Hospital, Weinan, China

**Keywords:** stress hyperglycemia ratio, atrial fibrillation, mortality, critical illness, prognosis, intensive care unit

## Abstract

**Background:**

The stress hyperglycemia ratio (SHR) has emerged as a potential prognostic indicator for various critical illnesses. However, its role in determining outcomes in patients with atrial fibrillation (AF) within the intensive care unit (ICU) remains unclear. This study aimed to elucidate the association between SHR and all-cause mortality in this clinical setting.

**Methods:**

We conducted a retrospective cohort study utilizing data from a large, retrospective database. Critically ill patients with documented AF were stratified based on quartiles of SHR. The primary outcome was 365-day all-cause mortality, with secondary outcomes including 90-day and 28-day mortality. COX proportional hazards models adjusted for confounders and Kaplan-Meier curve analyses were used to explore the relationship between SHR and mortality.

**Results:**

2,679 patients with critical AF were enrolled in the final study. Among the patients studied, those in the highest SHR quartiles exhibited an increased risk of 365-day all-cause mortality (HR:1.32, 95%CI=1.06-1.65). Notably, in subgroup analyses, the prognostic value of SHR was particularly pronounced in patients with hypertension. Sensitivity analyses confirmed the persistence of these findings after excluding cohorts with malignant tumors, and heart failure.

**Conclusions:**

Our research discerns a positive association between SHR and all-cause mortality in critically ill patients with AF, highlighting the significance of acute glycemic dysregulation on patient outcomes. Longer follow-up is still needed in the future to study the association between SHR and all-cause mortality in critically ill patients with AF.

## Introduction

Atrial fibrillation (AF), a substantial challenge in the field of cardiovascular treatment, affects approximately 60 million people globally (1). In recent years, the incidence of AF has been climbing steadily due to an aging population and increased awareness of the condition (2). As the most prevalent sustained cardiac arrhythmia, AF significantly heightens the risks of stroke, heart failure, and all-cause mortality (3–5). Evidence gathered from several countries asserts that the incidence of AF in the intensive care unit (ICU) hovers around one-sixth, and patients with AF experience a higher propensity for ischemic, thromboembolic, and severe bleeding events compared to non-AF patients (2). Given the high incidence and mortality of AF in critically ill ICU patients, pinpointing modifiable prognostic factors that can guide management decisions is critically important.

Among the myriad physiological changes occurring in critically ill patients, stress hyperglycemia ratio (SHR) is a widespread and consequential response, notably within the cardiology field (6, 7). A recent prospective cohort study from Asia involving 5,562 patients with acute coronary syndrome, with a two-year follow-up, discovered a J-shaped association between SHR and in-hospital cardiogenic death (8). Furthermore, a nationwide study from the United States similarly demonstrated that SHR was associated with all-cause mortality and cardiovascular mortality among patients with diabetes or prediabetes (9). Additionally, researchers have dissected the multifaceted roles of SHR, suggesting its involvement in both metabolic dysregulation and inflammatory pathways, potentially exacerbating cardiovascular complications (10, 11). Despite the emerging recognition of the prognostic relationship between SHR and adverse cardiovascular events such as myocardial infarction and heart failure, its direct association with the prognosis of patients with AF remains to be established (6, 12, 13).

Our study endeavors to bridge this knowledge gap by meticulously evaluating the impact of SHR on all-cause mortality among patients with AF admitted to the ICU. This investigation elucidates the role of SHR as a prognostic biomarker, enhancing clinicians’ understanding of patient stratification and management in the context of critical AF care.

## Methods

### Data source and study population

**Figure 1:**
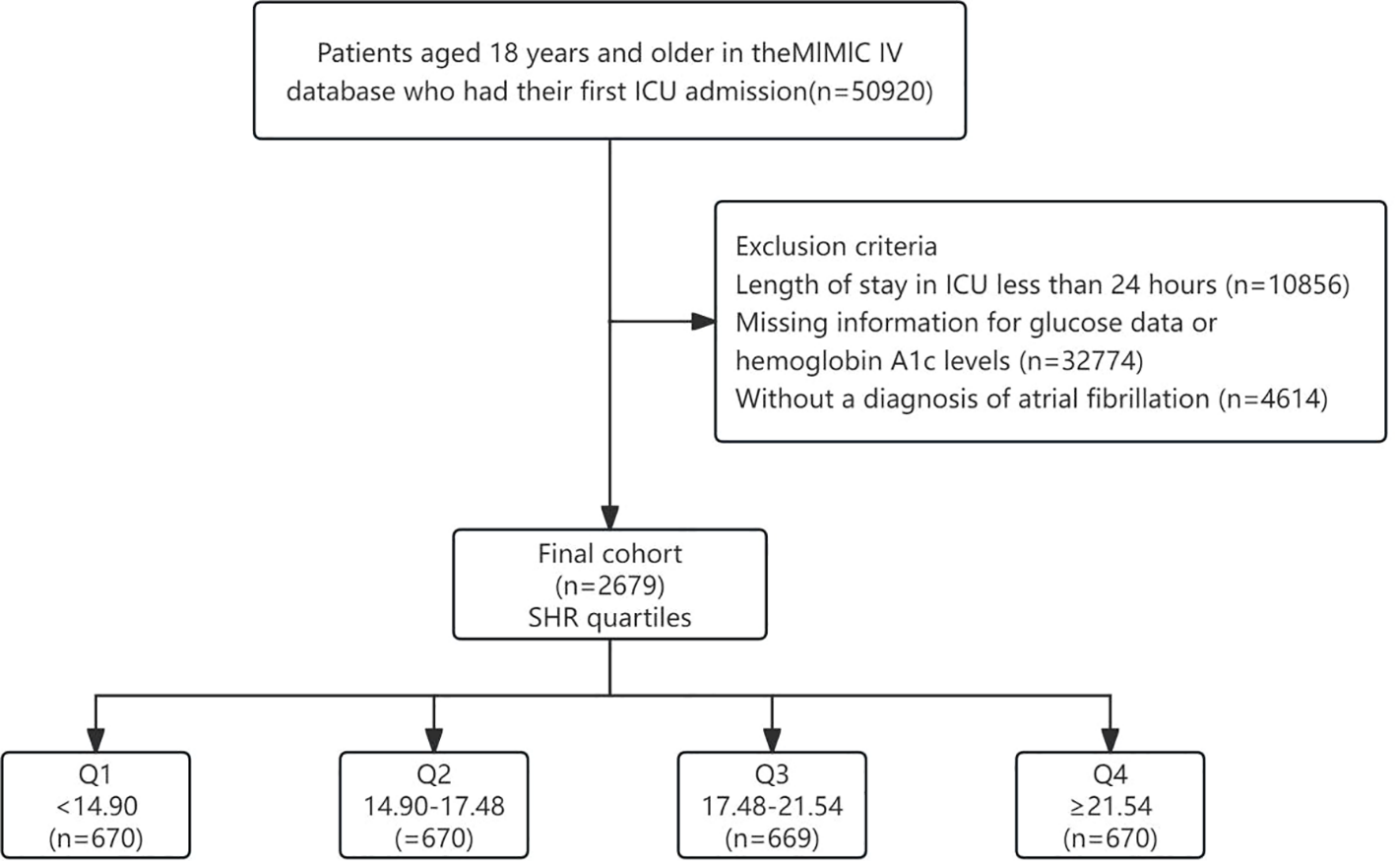
Flowchart of our study. SHR, Stress Hyperglycemia Ratio.

We conducted a retrospective cohort analysis using clinical data extracted from the Medical Information Mart for Intensive Care IV (MIMIC IV) database. MIMIC is an expansive, publicly available database containing de-identified health-related information of over 50,000 patients admitted to the ICU of the Beth Israel Deaconess Medical Center from 2008 to 2019. The author Fuqiang Liu was granted access to utilize this dataset (Record ID: 50081635). A total of 50,920 patients were initially included in our study. The inclusion criteria were: (1) Patients who were admitted to the ICU for the first time; (2) Patients aged 18 years or older. Exclusion criteria were as follows: (1) ICU stay shorter than 24 hours (n=10,856); (2) Absence of glucose data or hemoglobin A1c (HbA1c) levels (n=32774) at admission; (3) Patients without diagnosed with AF according to ICD-9 and ICD-10 codes (n=4614). Ultimately, a cohort of 2,679 patients were integrated into the study and were stratified according to SHR quartiles. The flowchart depicting the selection process of the study subjects is illustrated in **Figure 1**.

### Study variables

The primary exposure variable, SHR, was calculated by collecting admission blood glucose levels and HbA1c, using the formula: SHR = Admission blood glucose (mmol/L)/[(1.59 × HbA1c) - 2.59]. The primary outcome was defined as the all-cause mortality within 365 days following discharge. Secondary outcomes included all-cause mortality at 28 days and 90 days of discharge.

### Covariates

Demographic data, laboratory test results, and comorbidity information were extracted from the MIMIC-IV database using PostgreSQL (version 14.5). Demographics included age, gender, and weight. Laboratory tests encompassed measurements such as white blood cell (WBC), red blood cell (RBC), platelet, hemoglobin, red cell distribution width (RDW), hematocrit, sodium, potassium, chloride, anion gap, prothrombin time (PT), partial thromboplastin time (PTT), international normalized ratio (INR), urea nitrogen, and creatinine. Disease severity scoring systems, including SOFA, APS III, SAPS II, OASIS, and SIRS, were also incorporated into covariate analyses. Comorbidities considered in the study comprised conditions like hypertension, diabetes, chronic obstructive pulmonary disease (COPD), diabetes, heart failure, myocardial infarction (MI), stroke, hyperlipidemia, chronic kidney disease (CKD), malignant tumors, mechanical ventilation (MV), and vasoactive drugs.

For the management of missing values, continuous variables with more than 20% missing data were discarded. In contrast, for those with a smaller proportion of missing data, imputation was performed using the random forest method (R software, “*missForest”* package). Variables with missing information included in this study are shown in **Supplementary Table 1**.

### Statistical analyses

For categorical data, baseline variables were presented as frequencies and percentages, and differences were compared using the Chi-square test. Continuous variables were represented by means and standard deviations (SD), with differences evaluated using T-tests or one-way ANOVA as appropriate. Kaplan-Meier (KM) curves were used to compare mortality differences between various SHR groups, with the log-rank test employed for differential analyses. To quantify the relationship between SHR and all-cause mortality at 365 days, as well as at 28 and 90 days, COX proportional hazards regression analyses were utilized. Prior to using COX regression analyses, we tested the proportional risk assumption for the study variables using Schoenfeld residual plots, as well as calculating variance inflation factors (VIFs) to exclude variables with VIFs greater than 5. Adjustments for covariates were made in three models including Model 1 (unadjusted), Model 2 (adjusted for age, gender, and weight), and Model 3 (further adjusted for WBC, RBC, platelet, RDW, sodium, phosphate, anion gap, PT, PTT, SOFA, and history of hypertension, heart failure, COPD, MI, hyperlipidemia, MV, and vasoactive drugs), results are expressed as hazard ratios (HR) and 95% confidence intervals (95%CI). Restricted cubic splines (RCS) were employed to describe the dose-response relationship between SHR and mortality, with spline graphs created to visually delineate these relationships. Moreover, subgroup analyses were conducted to explore the influence of primary demographic and clinical characteristics on the potential effect. Finally, sensitivity analyses were performed to assess the robustness of our results by excluding patients with malignant tumors, and heart failure from the cohort.

All analyses were carried out using the R software (version 4.1.3, R Foundation for Statistical Computing, Vienna, Austria). A two-tailed P-value less than 0.05 was considered statistically significant.

## Results

### Baseline characteristics

**Table 1** provides a detailed exposition of the baseline characteristics of the 2,679 patients, stratified by SHR quartiles (Q1<14.90, 14.90≤Q2<17.48, 17.48≤Q3<21.54, 21.54≤Q4). The mean age of the participants was 74.15 years, and males constituted 61.52% of the population. Patients in the higher SHR quartiles tended to exhibit greater body weight, along with elevated levels of WBC, hemoglobin, hematocrit, anion gap, PTT, urea nitrogen, creatinine, and higher APS III, OASIS, and SIRS. Additionally, a higher proportion of patients in the upper SHR quartiles suffered from diabetes and MI and underwent MV treatment. Lengths of hospital stay, lengths of ICU stay, hospital mortality, ICU mortality, as well as 28-day, 90-day, and 365-day all-cause mortality were also highest in Q4. However, no significant differences were observed between groups about age, gender, RBC, platelet, RDW, sodium, phosphate, PT, INR, SOFA, SAPS II, or in patients with hypertension, COPD, heart failure, stroke, CKD, malignant tumors, and use of vasoactive drugs.

**Table 1:** Baseline characteristics of participants by SHR quartiles.

	Total (n = 2679)	SHR quartiles				
		Q1 (n = 670)	Q2 (n = 670)	Q3 (n = 669)	Q4 (n = 670)	P-value
Age, years	74.15 ± 10.49	74.21 ± 10.67	74.49 ± 10.55	74.50 ± 10.38	73.39 ± 10.33	0.168
Gender, n (%)						0.876
male	1648 (61.52)	408 (60.90)	421 (62.84)	408 (60.99)	411 (61.34)	
female	1031 (38.48)	262 (39.10)	249 (37.16)	261 (39.01)	259 (38.66)	
Weight, kg	85.00 ± 24.14	82.83 ± 24.07	86.36 ± 23.63	85.19 ± 24.88	85.63 ± 23.88	0.045
WBC, K/µL	12.81 ± 5.89	12.31 ± 6.51	12.72 ± 5.58	12.73 ± 5.47	13.49 ± 5.88	0.003
RBC, m/µL	3.55 ± 0.64	3.52 ± 0.59	3.58 ± 0.63	3.55 ± 0.66	3.56 ± 0.67	0.379
Platelet, K/µL	183.35 ± 78.41	179.25 ± 73.59	181.86 ± 76.43	182.05 ± 76.92	190.21 ± 85.91	0.060
Hemoglobin, g/dL	10.66 ± 1.86	10.46 ± 1.72	10.77 ± 1.80	10.68 ± 1.88	10.74 ± 2.02	0.008
RDW, %	14.70 ± 2.02	14.77 ± 2.00	14.57 ± 1.97	14.72 ± 2.04	14.75 ± 2.09	0.267
Hematocrit, %	32.25 ± 5.45	31.71 ± 5.07	32.52 ± 5.24	32.34 ± 5.56	32.43 ± 5.86	0.028
Sodium, mmol/L	138.30 ± 3.88	138.32 ± 3.82	138.54 ± 3.29	138.29 ± 3.96	138.07 ± 4.37	0.168
Potassium, mmol/L	4.31 ± 0.50	4.31 ± 0.49	4.29 ± 0.47	4.30 ± 0.53	4.32 ± 0.51	0.656
Chloride, mmol/L	104.80 ± 5.18	105.05 ± 5.25	105.31 ± 4.53	104.83 ± 4.94	104.03 ± 5.83	<0.001
Anion gap, mmol/L	13.51 ± 3.27	13.16 ± 3.36	13.22 ± 3.04	13.38 ± 3.16	14.28 ± 3.38	<0.001
PT, sec	15.78 ± 5.55	15.97 ± 5.73	15.79 ± 5.50	15.68 ± 5.67	15.69 ± 5.30	0.770
PTT, sec	38.87 ± 17.14	39.60 ± 18.05	38.00 ± 16.24	37.83 ± 15.15	40.06 ± 18.81	0.035
INR	1.45 ± 0.55	1.46 ± 0.54	1.45 ± 0.55	1.44 ± 0.60	1.43 ± 0.50	0.894
Urea nitrogen, mg/dL	24.92 ± 17.27	24.18 ± 17.73	23.07 ± 15.21	23.78 ± 15.24	28.63 ± 19.91	<0.001
Creatinine, mg/dL	1.28 ± 1.04	1.24 ± 1.01	1.20 ± 0.92	1.26 ± 1.03	1.42 ± 1.19	<0.001
SOFA	5.15 ± 3.14	5.08 ± 3.04	4.97 ± 3.04	5.17 ± 3.23	5.37 ± 3.24	0.122
APS III	43.38 ± 18.90	42.55 ± 19.92	40.46 ± 17.22	43.87 ± 18.48	46.65 ± 19.37	<0.001
SAPS II	39.25 ± 11.97	39.39 ± 12.40	38.24 ± 11.58	39.35 ± 11.91	40.01 ± 11.94	0.054
OASIS	32.83 ± 7.77	32.32 ± 7.66	32.02 ± 7.47	33.43 ± 7.77	33.56 ± 8.07	<0.001
SIRS	2.61 ± 0.90	2.51 ± 0.89	2.55 ± 0.93	2.63 ± 0.89	2.76 ± 0.86	<0.001
Hypertension, n (%)						0.178
no	1298 (48.45)	310 (46.27)	327 (48.81)	314 (46.94)	347 (51.79)	
yes	1381 (51.55)	360 (53.73)	343 (51.19)	355 (53.06)	323 (48.21)	
COPD, n (%)						0.266
no	2476 (92.42)	614 (91.64)	626 (93.43)	625 (93.42)	611 (91.19)	
yes	203 (7.58)	56 (8.36)	44 (6.57)	44 (6.58)	59 (8.81)	
Diabetes, n (%)						<0.001
no	1737 (64.84)	418 (62.39)	493 (73.58)	457 (68.31)	369 (55.07)	
yes	942 (35.16)	252 (37.61)	177 (26.42)	212 (31.69)	301 (44.93)	
Heart failure, n (%)						0.078
no	1542 (57.56)	395 (58.96)	392 (58.51)	398 (59.49)	357 (53.28)	
yes	1137 (42.44)	275 (41.04)	278 (41.49)	271 (40.51)	313 (46.72)	
MI, n (%)						<0.001
no	2309 (86.19)	602 (89.85)	607 (90.60)	575 (85.95)	525 (78.36)	
yes	370 (13.81)	68 (10.15)	63 (9.40)	94 (14.05)	145 (21.64)	
Stroke, n (%)						0.297
no	2276 (84.96)	558 (83.28)	563 (84.03)	577 (86.25)	578 (86.27)	
yes	403 (15.04)	112 (16.72)	107 (15.97)	92 (13.75)	92 (13.73)	
Hyperlipidemia, n (%)						0.001
no	1225 (45.73)	275 (41.04)	311 (46.42)	294 (43.95)	345 (51.49)	
yes	1454 (54.27)	395 (58.96)	359 (53.58)	375 (56.05)	325 (48.51)	
CKD, n (%)						0.821
no	2102 (78.46)	528 (78.81)	530 (79.10)	527 (78.77)	517 (77.16)	
yes	577 (21.54)	142 (21.19)	140 (20.90)	142 (21.23)	153 (22.84)	
Malignant tumors, n (%)						0.345
no	2251 (84.02)	570 (85.07)	563 (84.03)	548 (81.91)	570 (85.07)	
yes	428 (15.98)	100 (14.93)	107 (15.97)	121 (18.09)	100 (14.93)	
MV, n (%)						0.026
no	275 (10.27)	77 (11.49)	82 (12.24)	65 (9.72)	51 (7.61)	
yes	2404 (89.73)	593 (88.51)	588 (87.76)	604 (90.28)	619 (92.39)	
Vasoactive drugs, n (%)						0.235
no	1113 (41.55)	278 (41.49)	257 (38.36)	289 (43.20)	289 (43.13)	
yes	1566 (58.45)	392 (58.51)	413 (61.64)	380 (56.80)	381 (56.87)	
LOS of hospital, day	12.33 ± 10.38	12.31 ± 12.44	11.63 ± 9.38	11.92 ± 8.64	13.47 ± 10.58	0.007
LOS of ICU, day	4.62 ± 5.84	4.15 ± 5.81	4.29 ± 5.64	4.76 ± 5.70	5.27 ± 6.13	0.002
Hospital mortality, n (%)						<0.001
no	2448 (91.38)	618 (92.24)	632 (94.33)	611 (91.33)	587 (87.61)	
yes	231 (8.62)	52 (7.76)	38 (5.67)	58 (8.67)	83 (12.39)	
ICU mortality, n (%)						0.008
no	2540 (94.81)	640 (95.52)	649 (96.87)	626 (93.57)	625 (93.28)	
yes	139 (5.19)	30 (4.48)	21 (3.13)	43 (6.43)	45 (6.72)	
28-day mortality, n (%)						<0.001
no	2376 (88.69)	602 (89.85)	617 (92.09)	593 (88.64)	564 (84.18)	
yes	303 (11.31)	68 (10.15)	53 (7.91)	76 (11.36)	106 (15.82)	
90-day mortality, n (%)						<0.001
no	2244 (83.76)	565 (84.33)	595 (88.81)	559 (83.56)	525 (78.36)	
yes	435 (16.24)	105 (15.67)	75 (11.19)	110 (16.44)	145 (21.64)	
365-day mortality, n (%)						<0.001
no	2095 (78.20)	525 (78.36)	559 (83.43)	528 (78.92)	483 (72.09)	
yes	584 (21.80)	145 (21.64)	111 (16.57)	141 (21.08)	187 (27.91)	

SHR, Stress Hyperglycemia Ratio; WBC, White Blood Cell; RBC, Red Blood Cell; RDW, Red cell Distribution Width; PT, Prothrombin Time; PTT, Partial Thromboplastin Time; INR, International Normalized Ratio; SOFA, Sequential Organ Failure Assessment. APS III, Acute Physiology Score III; SAPS II, Simplified Acute Physiology Score; OASIS, Oxford Acute Severity of Illness Score; SIRS, Systemic Inflammatory Response Syndrome; COPD, Chronic Obstructive Pulmonary Disease; MI, Myocardial Infarction; CKD, Chronic Kidney Disease; MV, Mechanical Ventilation; LOS, Length of Stay.

Continuous variables are expressed as mean (SD) and categorical variables are expressed as frequency (percentage).

**Table 2** outlines the baseline characteristics at 365 days for both survival and non-survival cohorts. There were 2,095 in the survivor group and 584 in the non-survivor group. The non-survival group typically exhibited higher values of SHR, age, proportion of female, platelet, RDW, anion gap, PT, PTT, INR, urea nitrogen, creatinine, and higher SOFA, APS III, SAPS II, and OASIS. Survivors often had higher levels of hemoglobin, and chloride, and higher rates of MV and use of vasoactive drugs. Additionally, survivors also tended to have higher body weight and a greater prevalence of hypertension and hyperlipidemia. Lower incidences of COPD, heart failure, malignant tumors, CKD, and stroke were observed among survivors.

**Table 2:** Baseline characteristics of survivor and non-survivor groups.

	Total (n = 2679)	survival (n = 2095)	Non-survival (n = 584)	P-value
SHR	19.08 ± 6.82	18.78 ± 6.43	20.17 ± 8.00	<0.001
Age, years	74.15 ± 10.49	72.87 ± 10.08	78.71 ± 10.65	<0.001
Gender, n (%)				<0.001
male	1648 (61.52)	1354 (64.63)	294 (50.34)	
female	1031 (38.48)	741 (35.37)	290 (49.66)	
Weight, kg	85.00 ± 24.14	86.90 ± 23.24	78.20 ± 26.04	<0.001
WBC, K/µL	12.81 ± 5.89	12.87 ± 5.44	12.62 ± 7.29	0.452
RBC, m/µL	3.55 ± 0.64	3.55 ± 0.62	3.55 ± 0.71	0.809
Platelet, K/µL	183.35 ± 78.41	179.17 ± 74.86	198.32 ± 88.48	<0.001
Hemoglobin, g/dL	10.66 ± 1.86	10.71 ± 1.81	10.51 ± 2.02	0.036
RDW, %	14.70 ± 2.02	14.42 ± 1.73	15.72 ± 2.59	<0.001
Hematocrit, %	32.25 ± 5.45	32.23 ± 5.29	32.32 ± 5.98	0.740
Sodium, mmol/L	138.30 ± 3.88	138.25 ± 3.52	138.51 ± 4.95	0.225
Potassium, mmol/L	4.31 ± 0.50	4.31 ± 0.46	4.30 ± 0.63	0.960
Chloride, mmol/L	104.80 ± 5.18	105.22 ± 4.77	103.30 ± 6.19	<0.001
Anion gap, mmol/L	13.51 ± 3.27	13.02 ± 3.03	15.27 ± 3.48	<0.001
PT, sec	15.78 ± 5.55	15.29 ± 4.47	17.55 ± 8.11	<0.001
PTT, sec	38.87 ± 17.14	37.88 ± 15.92	42.42 ± 20.57	<0.001
INR	1.45 ± 0.55	1.40 ± 0.42	1.62 ± 0.83	<0.001
Urea nitrogen, mg/dL	24.92 ± 17.27	22.47 ± 14.96	33.68 ± 21.61	<0.001
Creatinine, mg/dL	1.28 ± 1.04	1.18 ± 0.93	1.63 ± 1.33	<0.001
SOFA	5.15 ± 3.14	5.02 ± 3.05	5.61 ± 3.42	<0.001
APS III	43.38 ± 18.90	40.95 ± 18.07	52.10 ± 19.26	<0.001
SAPS II	39.25 ± 11.97	38.04 ± 11.75	43.58 ± 11.77	<0.001
OASIS	32.83 ± 7.77	32.03 ± 7.56	35.71 ± 7.83	<0.001
SIRS	2.61 ± 0.90	2.59 ± 0.89	2.67 ± 0.91	0.051
Hypertension, n (%)				<0.001
no	1298 (48.45)	962 (45.92)	336 (57.53)	
yes	1381 (51.55)	1133 (54.08)	248 (42.47)	
COPD, n (%)				0.002
no	2476 (92.42)	1954 (93.27)	522 (89.38)	
yes	203 (7.58)	141 (6.73)	62 (10.62)	
Diabetes, n (%)				0.215
no	1737 (64.84)	1371 (65.44)	366 (62.67)	
yes	942 (35.16)	724 (34.56)	218 (37.33)	
Heart failure, n (%)				<0.001
no	1542 (57.56)	1261 (60.19)	281 (48.12)	
yes	1137 (42.44)	834 (39.81)	303 (51.88)	
MI, n (%)				0.855
no	2309 (86.19)	1807 (86.25)	502 (85.96)	
yes	370 (13.81)	288 (13.75)	82 (14.04)	
Malignant tumors, n (%)				0.001
no	2251 (84.02)	1786 (85.25)	465 (79.62)	
yes	428 (15.98)	309 (14.75)	119 (20.38)	
CKD, n (%)				<0.001
no	2102 (78.46)	1705 (81.38)	397 (67.98)	
yes	577 (21.54)	390 (18.62)	187 (32.02)	
Stroke, n (%)				<0.001
no	2276 (84.96)	1816 (86.68)	460 (78.77)	
yes	403 (15.04)	279 (13.32)	124 (21.23)	
Hyperlipidemia, n (%)				0.003
no	1225 (45.73)	926 (44.20)	299 (51.20)	
yes	1454 (54.27)	1169 (55.80)	285 (48.80)	
MI, n (%)				0.796
no	2307 (86.11)	1806 (86.21)	501 (85.79)	
yes	372 (13.89)	289 (13.79)	83 (14.21)	
MV, n (%)				0.020
no	275 (10.27)	200 (9.55)	75 (12.84)	
yes	2404 (89.73)	1895 (90.45)	509 (87.16)	
Vasoactive drugs, n (%)				<0.001
no	1113 (41.55)	824 (39.33)	289 (49.49)	
yes	1566 (58.45)	1271 (60.67)	295 (50.51)	

SHR, Stress Hyperglycemia Ratio; WBC, White Blood Cell; RBC, Red Blood Cell; RDW, Red cell Distribution Width; PT, Prothrombin Time; PTT, Partial Thromboplastin Time; INR, International Normalized Ratio; SOFA, Sequential Organ Failure Assessment. APS III, Acute Physiology Score III; SAPS II, Simplified Acute Physiology Score; OASIS, Oxford Acute Severity of Illness Score; SIRS, Systemic Inflammatory Response Syndrome; MI, Myocardial Infarction; COPD, Chronic Obstructive Pulmonary Disease; CKD, Chronic Kidney Disease; MV, Mechanical Ventilation.

Continuous variables are expressed as mean (SD) and categorical variables are expressed as frequency (percentage).

### Association between SHR and mortality in critically ill patients with AF

**Figure 2:**
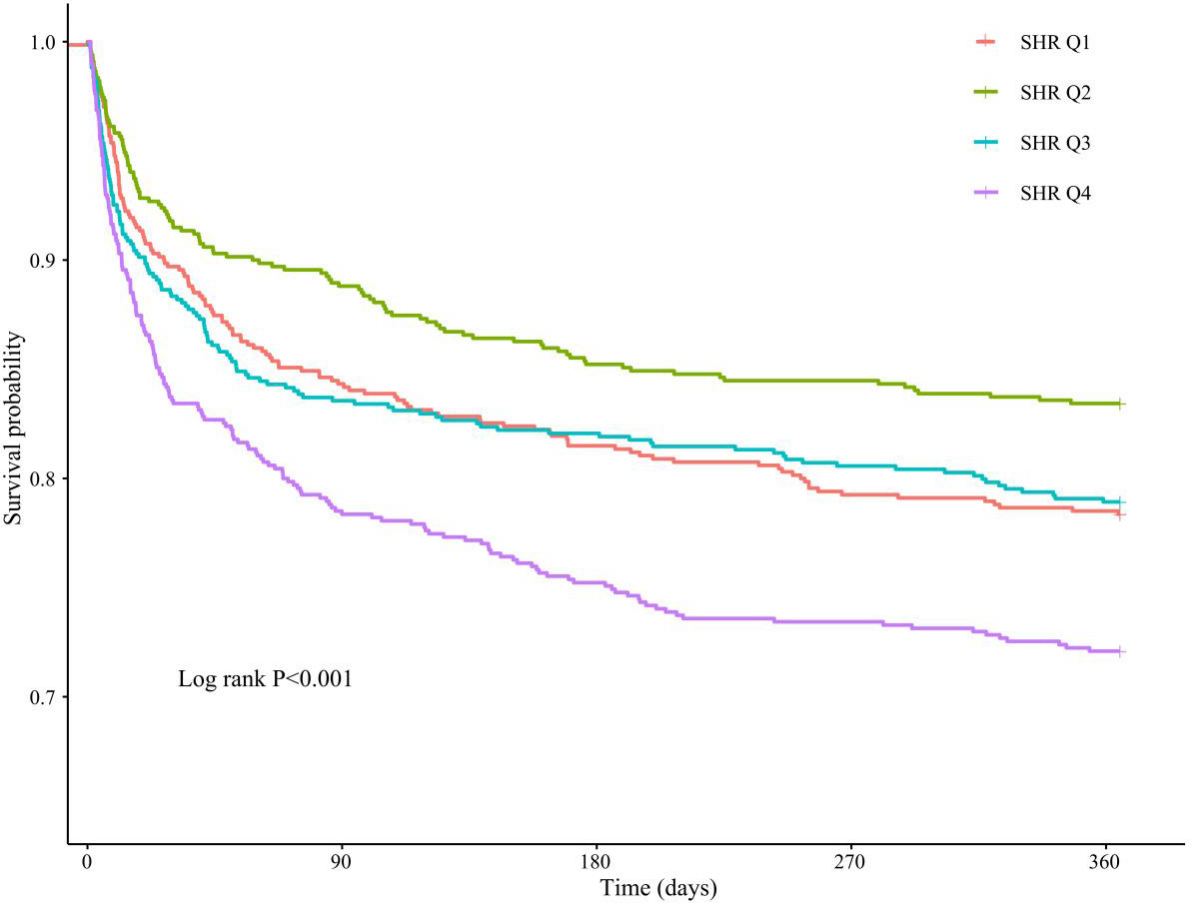
KM analysis between SHR quartiles and 365-day all-cause mortality in critically ill patients with AF.

KM curve analyses revealed that there were statistically significant differences in 365-day all-cause mortality among patients stratified by quartiles of SHR (log-rank P<0.001), with significantly lower mortality rates in the Q1, Q2, and Q3 quartiles compared with Q4 (**Figure 2**). A similar statistical significance was observed between SHR quartiles in terms of 90-day and 28-day all-cause mortality (**Supplementary Figure 1**).

In **Table 3**, the relationship between SHR and the 365-day all-cause mortality among critically ill patients with AF was evaluated using a multivariate COX proportional hazards model. In the fully adjusted model, for each unit increase in SHR, the risk of 365-day all-cause mortality increased by 3% (HR=1.03; 95%CI: 1.02-1.04; P < 0.001). When divided by quartiles of SHR and taking Q1 as the reference, the risk was significantly lower in Q2 (HR=0.74; 95%CI: 0.58-0.95; P = 0.019), not significantly different in Q3 (HR=0.98; 95CI: 0.78-1.24; P = 0.885), and significantly higher in Q4 (HR=1.36; 95%CI: 1.09-1.68; P = 0.006), demonstrating a significant trend across the quartiles (P for trend < 0.001). Additionally, in studies of SHR with 28-day and 90-day all-cause mortality, a similar pattern was observed where an increase in SHR levels was associated with an increased all-cause mortality (**Supplementary Table 2**).

**Table 3:** Association between SHR and 365-d all-cause mortality in critically ill patients with AF.

	Model 1		Model 2		Model 3	
	HR (95%CI)	P-value	HR (95%CI)	P-value	HR (95%CI)	P-value
SHR	1.03(1.02,1.04)	<0.001	1.03(1.02,1.04)	<0.001	1.02(1.01,1.03)	0.002
SHR quartiles						
Q1	Ref		Ref		Ref	
Q2	0.74(0.58,0.95)	0.019	0.75(0.59,0.96)	0.023	0.81(0.63,1.04)	0.099
Q3	0.98(0.78,1.24)	0.885	0.97(0.77,1.23)	0.811	1.02(0.81,1.29)	0.875
Q4	1.36(1.09,1.68)	0.006	1.40(1.12,1.74)	0.003	1.32(1.06,1.65)	0.015
P for trend		<0.001		<0.001		0.006

Model 1: no adjusted.

Model 2: adjusted for age, gender, weight, WBC, RBC, and platelet.

Model 3: adjusted for age, gender, weight, WBC, RBC, platelet, RDW, sodium, phosphorus, anion gap, PT, PTT, SOFA, hypertension, heart failure, COPD, MI, hyperlipidemia, MV, and vasoactive drug.

HR, Hazard Ratio; CI, Confidence Interval; Ref, Reference.

**Figure 3:**
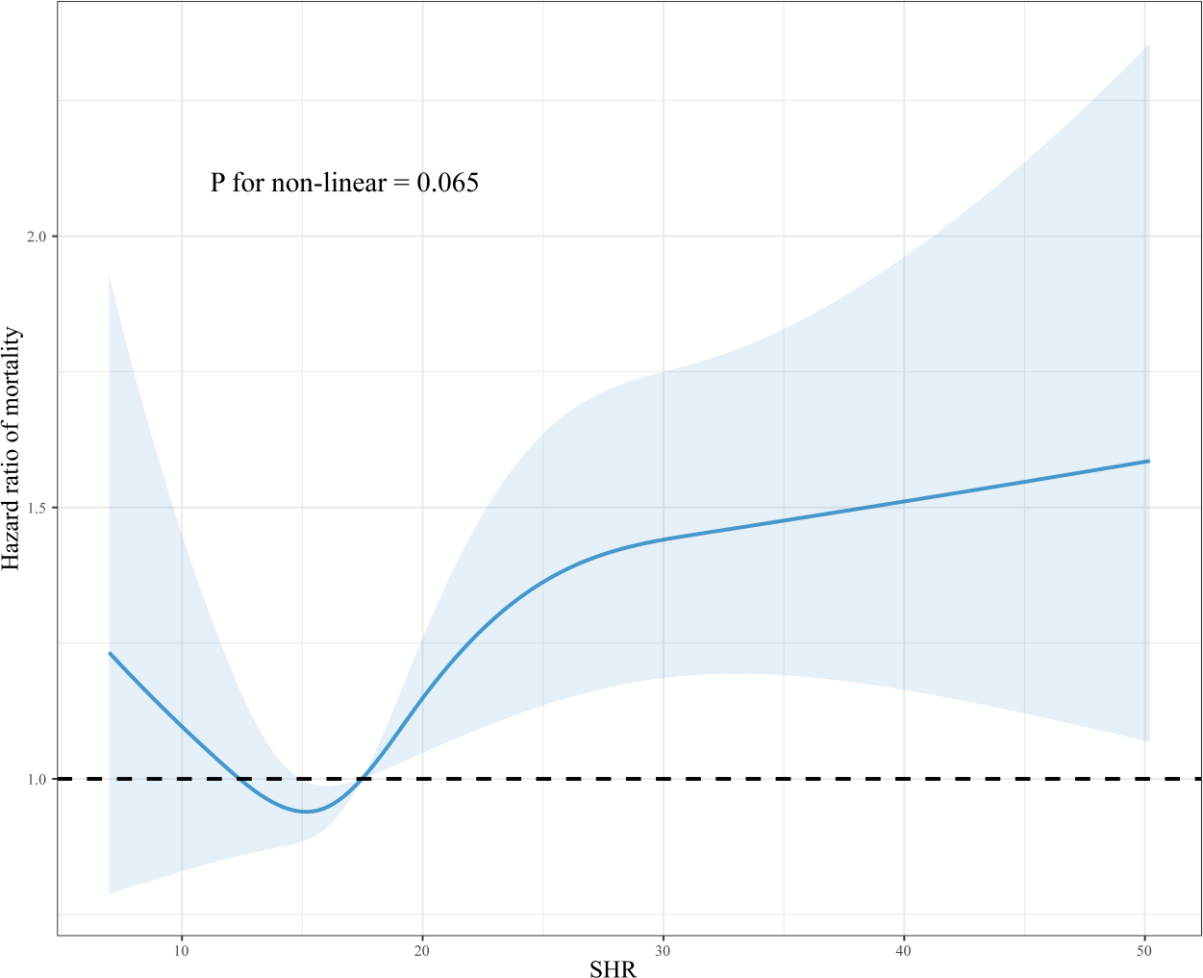
Dose-response association between SHR and 365-day all-cause mortality in critically ill patients with AF. RCS was adjusted for age, gender, weight, WBC, RBC, platelet, RDW, sodium, phosphorus, anion gap, PT, PTT, SOFA, hypertension, heart failure, COPD, MI, hyperlipidemia, MV, and vasoactive drugs.

Treating SHR as a continuous variable, the RCS model revealed its relationship with all-cause mortality within 365 days. After adjusting for confounders, the 365-day all-cause mortality increased with increasing levels of SHR, although this trend was decreasing at the outset but did not constitute statistical significance (P for non-linear>0.05) (**Figure 3**). However, in the studies of 28-day and 90-day all-cause mortality, a non-linear positive association between SHR and mortality was observed (P for non-linear<0.05) (**Supplementary Figure 2**).

### Subgroup analyses

**Figure 4:**
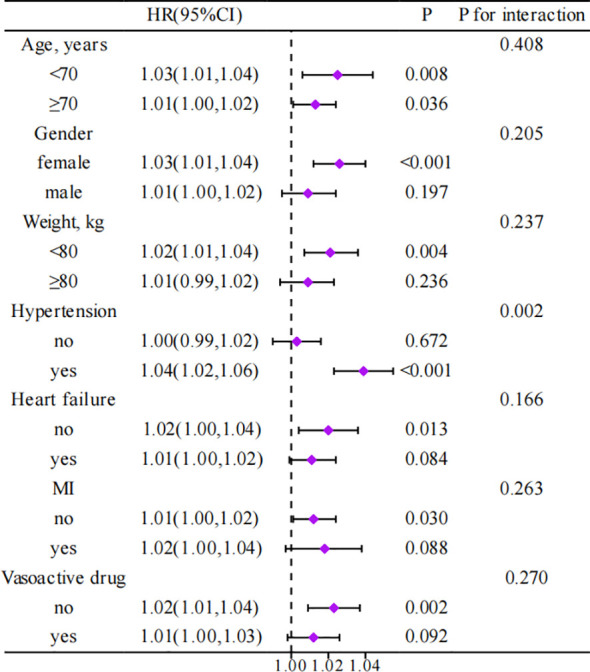
Association between different characteristics of SHR and 365-day all-cause mortality in critically ill patients with AF. HR, Hazard Ratio; CI, Confidence Interval; MI, Myocardial Infarction.

**Figure 4** presents a subgroup analysis investigating the relationship between SHR and 365-day all-cause mortality among critically ill patients with different characteristics, as well as whether the characteristic alters this relationship. Although HRs vary across different patient subgroups, there is a general positive association between SHR and 365-day all-cause mortality. Interactions revealed that age, gender, body weight, heart failure, MI, and the use of vasoactive drugs do not affect this association. Conversely, hypertension significantly interacts with SHR, influencing the impact on mortality.

### Sensitivity analyses

The sensitivity analyses were conducted to clarify the robustness of the relationship between SHR and the 365-day all-cause mortality among critically ill patients with AF after excluding those with malignant tumors. The results persistently indicated that higher SHR levels are significantly associated with an increased risk of mortality. Furthermore, the analyses were extended by excluding patients with heart failure, and the findings consistently demonstrated a positive association between SHR and 365-day all-cause mortality among critically ill patients with AF (**Supplementary Tables 3**-**4**).

## Discussion

To our knowledge, this is the first study to explore the relationship between SHR and all-cause mortality in critically ill patients with AF. Our findings indicate a positive association between SHR and all-cause mortality, with higher SHR levels associated with increased mortality at 365 days, 90 days, and 28 days.

In recent years, as the incidence of diabetes has continued to rise, SHR has garnered increasing attention from researchers. SHR has been proven in the cardiovascular field to be a strong predictor of patient mortality. Zeng et al. reported a significant association between SHR and all-cause mortality in patients with acute coronary syndrome over a median follow-up of 2.1 years, with the highest tertile of SHR increasing the risk of death by 80% (14). Additionally, Qing et al. documented patients with acute decompensated heart failure at the highest SHR range had significantly increased risks of all-cause and cardiovascular mortality (6). SHR has also been shown to predict outcomes effectively in diseases such as stroke, aortic stenosis, and diabetes (6, 15, 16). Consistent with previous studies, our research, after adjusting for potential confounders, found that higher SHR is associated with increased all-cause mortality in critically ill patients with AF.

Although our study did not further elucidate the mechanisms underlying the association between SHR and all-cause mortality in critically ill patients with AF, we consider several factors that may play an important role. Firstly, SHR has been found to exacerbate the inflammatory cascade, increase oxidative stress, and impair vascular function, all of which significantly impact patient prognosis (17–19). AF itself is associated with systemic inflammation and thrombosis, and the resultant dysregulation caused by the overlay of high SHR states may exacerbate the risk of death (20, 21). In addition, higher blood sugar levels can alter ionic currents, leading to further cardiac arrhythmia and an increase in mortality (22). Finally, higher levels of SHR can adversely affect cardiomyocyte function and myocardial energy production, leading to aggravated AF and an increased risk of death (23, 24). Biologically, glycosylation end products formed by hyperglycemia may lead to fibrosis and increased atrial stiffness (25). In addition, stress hyperglycemia also induces an adrenergic response, which is associated with left atrial dilatation (26). Whereas, increased atrial stiffness and left atrial remodeling may be the main factors contributing to the development of atrial fibrillation (27). In addition, AF itself may have potential feedback effects on SHR.AF may lead to increased cardiac load and altered cardiac function, and these changes may indirectly affect blood glucose levels and SHR by affecting metabolic pathways and insulin signaling (28).

The results of our subgroup analyses offered deeper insight into the prognostic significance of SHR among patients with various characteristics. Interestingly, the predictive value of SHR differed between hypertensive and non-hypertensive groups. In critically ill AF patients with hypertension, the impact of SHR on all-cause mortality was more pronounced, suggesting an increased need to focus on patients with concomitant hypertension. Additionally, our sensitivity analyses confirmed the robustness of this association. By excluding patients with malignant tumors, and heart failure, we ascertained that the observed relationship was not unduly influenced by these conditions, which could independently affect SHR and survival outcomes.

Our study utilized a large and well-characterized database to reveal the association between SHR and all-cause mortality in critically ill patients with AF. Nonetheless, several limitations should be considered when interpreting our results. Firstly, as we utilized the MIMIC-IV database, analyses were limited to pre-collected data. Despite adjusting for multiple variables, residual confounding factors not included in the database may affect our findings. Secondly, the MIMIC-IV database reflects a single-center experience, potentially limiting the generalizability of our findings to broader populations and healthcare systems. Patients treated at Beth Israel Deaconess Medical Center may not fully represent the demographic or clinical variability of other regions or nations. Thirdly, SHR was calculated based on admission glucose concentration and HbA1c, which could entail measurement biases, and future studies should consider multiple measurements and calculations of SHR, including the variability of SHR in their scope. Fourth, Since the study focuses on ICU patients, the applicability of this indicator to outpatient settings is limited. For ICU patients, SHR is highly relevant, but its utility in outpatient care is minimal. Future research should explore the applicability of SHR in other clinical settings or develop alternative methods for assessing stress hyperglycemia in outpatient populations. In addition, further exploration of interventions to reduce SHR is necessary for special populations.

## Conclusion

In summary, this study highlights the prognostic potential of SHR in critically ill patients with AF, unveiling a positive association between SHR and all-cause mortality and simultaneously underscoring the particularities within the hypertensive population. Our results lay the groundwork for future prospective studies and clinical trials.

## Data Availability

The raw data supporting the conclusions of this article will be made available by the authors, without undue reservation.
