# Supplementary material for: Association between stress hyperglycemia ratio and all-cause mortality in critically ill patients with atrial fibrillation: insights from a MIMIC-IV study
Source: Front Endocrinol (Lausanne). 2024 Aug 23;15:1412159. doi: 10.3389/fendo.2024.1412159 (PMC11377218; doi:10.3389/fendo.2024.1412159)
Supplement: Supplementary file 1 [file DataSheet1.doc]

**Supplementary Figures and Tables:**

Supplementary Figure 1. KM analyses for 90-day and 28-day all-cause mortality between SHR quartiles

Supplementary Figure 2. Dose-response association between SHR and 90-day and 28-day all-cause mortality

Supplementary Table 1. Missing values of the variables included in our study

Supplementary Table 2. Association between SHR and all-cause mortality in critically ill patients with AF

Supplementary Table 3. Sensitivity analysis between SHR and 365-day all-cause mortality in patients with excluded malignant tumor

Supplementary Table 4. Sensitivity analysis between SHR and 365-day all-cause mortality in patients with excluded heart failure


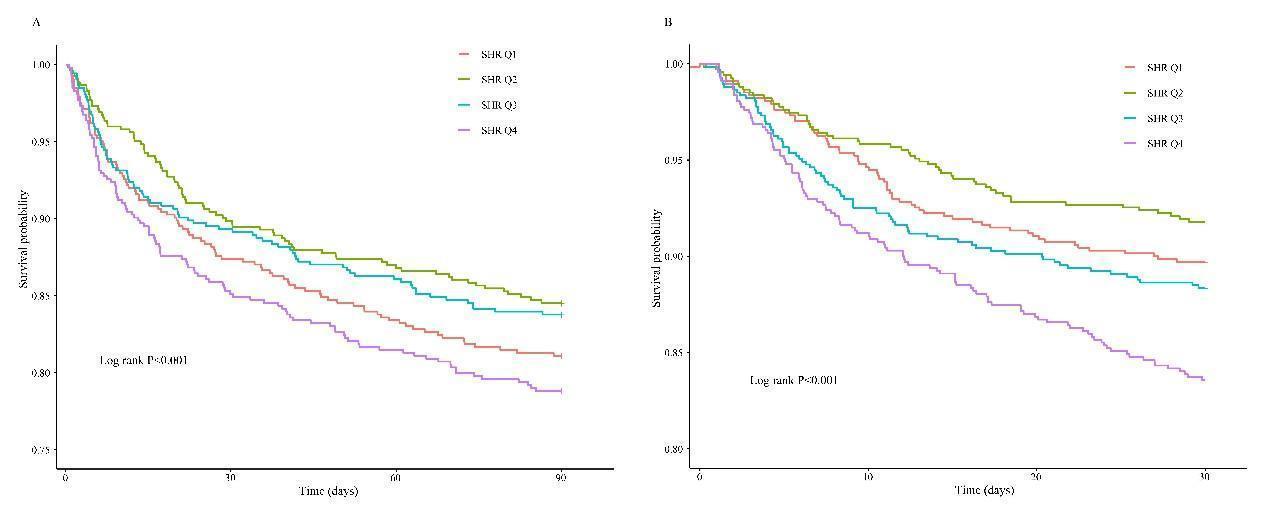


Supplementary Figure 1. KM analyses for 90-day and 28-day all-cause mortality between SHR quartiles. (A: 90-day all-cause mortality; B: 28-day all-cause mortality)


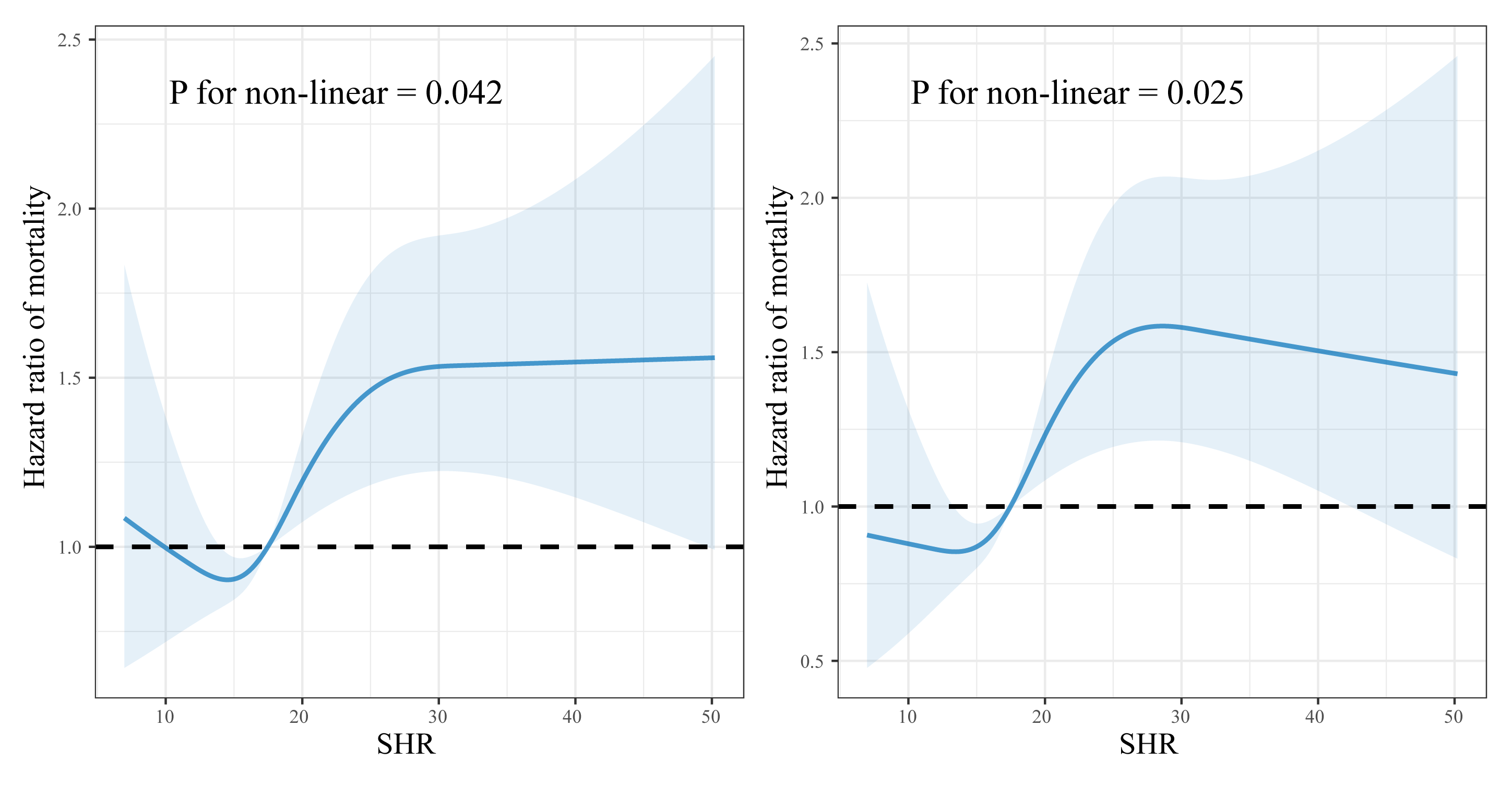


Supplementary Figure 2. Dose-response association between SHR and 90-day and 28-day all-cause mortality. RCS were adjusted for age, gender, weight, WBC, RBC, platelet, RDW, sodium, phosphorus, anion gap, PT, PTT, SOFA, hypertension, heart failure, COPD, MI, hyperlipidemia, MV, and vasoactive drug. (A: 90-day all-cause mortality; B: 28-day all-cause mortality)

| Supplementary Table 1. Missing values of the variables included in our study | | |
| --- | --- | --- |
| Variables | Missing patients | Complete rate |
| WBC | 13 | 0.995 |
| RBC | 13 | 0.995 |
| Platelet | 13 | 0.995 |
| Hemoglobin | 13 | 0.995 |
| RDW | 13 | 0.995 |
| Hematocrit | 13 | 0.995 |
| Sodium | 14 | 0.995 |
| Potassium | 17 | 0.994 |
| Chloride | 12 | 0.996 |
| Anion gap | 25 | 0.991 |
| PT | 103 | 0.962 |
| PTT | 109 | 0.959 |
| INR | 103 | 0.962 |
| Urea nitrogen | 14 | 0.995 |
| Creatinine | 12 | 0.996 |
| WBC, White Blood Cell; RBC, Red Blood Cell; RDW, Red cell Distribution Width; PT, Prothrombin Time; PTT, Partial Thromboplastin Time; INR, International Normalized Ratio. | | |

| Supplementary Table 2. Association between SHR and all-cause mortality in critically ill patients with AF | | | | | | |
| --- | --- | --- | --- | --- | --- | --- |
|  | **Model 1** | | **Model 2** | | **Model 3** | |
|  | HR (95%CI) | P-value | HR (95%CI) | P-value | HR (95%CI) | P-value |
| **90-day** |  |  |  |  |  |  |
| SHR | 1.03(1.02,1.04) | <0.001 | 1.03(1.02,1.04) | <0.001 | 1.02(1.01,1.03) | 0.001 |
| SHR quartiles |  |  |  |  |  |  |
| Q1 | Ref |  | Ref |  | Ref |  |
| Q2 | 0.70(0.52,0.94) | 0.019 | 0.70(0.52,0.95) | 0.021 | 0.79(0.59,1.07) | 0.128 |
| Q3 | 1.06(0.81,1.39) | 0.650 | 1.05(0.81,1.38) | 0.700 | 1.15(0.87,1.50) | 0.325 |
| Q4 | 1.44(1.12,1.85) | 0.005 | 1.46(1.13,1.87) | 0.004 | 1.42(1.10,1.84) | 0.007 |
| P for trend |  | 0.002 |  | 0.001 |  | 0.031 |
| **28-day** |  |  |  |  |  |  |
| SHR | 1.03(1.02,1.04) | <0.001 | 1.03(1.02,1.04) | <0.001 | 1.02(1.01,1.03) | 0.002 |
| SHR quartiles |  |  |  |  |  |  |
| Q1 | Ref |  | Ref |  | Ref |  |
| Q2 | 0.79(0.55,1.12) | 0.187 | 0.78(0.55,1.11) | 0.169 | 0.90(0.63,1.30) | 0.581 |
| Q3 | 1.15(0.83,1.59) | 0.404 | 1.12(0.81,1.55) | 0.485 | 1.23(0.88,1.71) | 0.217 |
| Q4 | 1.65(1.22,2.22) | 0.001 | 1.64(1.21,2.22) | 0.001 | 1.64(1.20,2.24) | 0.002 |
| P for trend |  | <0.001 |  | <0.001 |  | <0.001 |
| Model 1: no adjusted;  Model 2: adjusted for age, gender, weight, WBC, RBC, and platelet;  Model 3: adjusted for age, gender, weight, WBC, RBC, platelet, RDW, sodium, phosphorus, anion gap, PT, PTT, SOFA, hypertension, heart failure, COPD, MI, hyperlipidemia, MV, and vasoactive drug.  HR, Hazard Ratio; CI, Confidence Interval; Ref, Reference. | | | | | | |

| Supplementary Table 3. Sensitivity analysis between SHR and 365-day all-cause mortality in patients with excluded malignant tumor | | | | | | |
| --- | --- | --- | --- | --- | --- | --- |
|  | **Model 1** | | **Model 2** | | **Model 3** | |
|  | HR (95%CI) | P-value | HR (95%CI) | P-value | HR (95%CI) | P-value |
| SHR | 1.03(1.02,1.04) | <0.001 | 1.03(1.02,1.04) | <0.001 | 1.02(1.01,1.03) | 0.001 |
| SHR quartiles |  |  |  |  |  |  |
| Q1 | Ref |  | Ref |  | Ref |  |
| Q2 | 0.67(0.51,0.89) | 0.005 | 0.69(0.52,0.91) | 0.010 | 0.78(0.58,1.03) | 0.081 |
| Q3 | 0.91(0.70,1.19) | 0.488 | 0.92(0.70,1.19) | 0.507 | 0.98(0.75,1.28) | 0.898 |
| Q4 | 1.38(1.09,1.75) | 0.007 | 1.44(1.13,1.83) | 0.003 | 1.39(1.09,1.77) | 0.008 |
| P for trend |  | <0.001 |  | <0.001 |  | 0.001 |
| Model 1: no adjusted;  Model 2: adjusted for age, gender, weight, WBC, RBC, and platelet;  Model 3: adjusted for age, gender, weight, WBC, RBC, platelet, RDW, sodium, phosphorus, anion gap, PT, PTT, SOFA, hypertension, heart failure, COPD, MI, hyperlipidemia, MV, and vasoactive drug.  HR, Hazard Ratio; CI, Confidence Interval; Ref, Reference. | | | | | | |

| Supplementary Table 4. Sensitivity analysis between SHR and 365-day all-cause mortality in patients with excluded heart failure | | | | | | |
| --- | --- | --- | --- | --- | --- | --- |
|  | **Model 1** | | **Model 2** | | **Model 3** | |
|  | HR (95%CI) | P-value | HR (95%CI) | P-value | HR (95%CI) | P-value |
| SHR | 1.03(1.02,1.05) | <0.001 | 1.04(1.02,1.05) | <0.001 | 1.02(1.00,1.04) | 0.010 |
| SHR quartiles |  |  |  |  |  |  |
| Q1 | Ref |  | Ref |  | Ref |  |
| Q2 | 0.88(0.61,1.26) | 0.479 | 0.91(0.63,1.31) | 0.612 | 0.91(0.63,1.31) | 0.607 |
| Q3 | 1.22(0.88,1.71) | 0.237 | 1.26(0.90,1.76) | 0.182 | 1.17(0.83,1.65) | 0.365 |
| Q4 | 1.65(1.20,2.29) | 0.002 | 1.80(1.30,2.49) | <0.001 | 1.49(1.06,2.08) | 0.021 |
| P for trend |  | 0.042 |  | 0.025 |  | 0.115 |
| Model 1: no adjusted;  Model 2: adjusted for age, gender, weight, WBC, RBC, and platelet;  Model 3: adjusted for age, gender, weight, WBC, RBC, platelet, RDW, sodium, phosphorus, anion gap, PT, PTT, SOFA, hypertension, COPD, MI, hyperlipidemia, MV, and vasoactive drug.  HR, Hazard Ratio; CI, Confidence Interval; Ref, Reference. | | | | | | |
